# Lower Expression of TWEAK is Associated with Poor Survival and Dysregulate TIICs in Lung Adenocarcinoma

**DOI:** 10.1155/2022/8661423

**Published:** 2022-06-06

**Authors:** Zhengxi He, Sai Wang, Jinchun Wu, Yangchun Xie, Bin Li

**Affiliations:** ^1^Department of Oncology, Xiangya Hospital, Central South University, Changsha, Hunan 410008, China; ^2^Hunan Cancer Hospital and The Affiliated Cancer Hospital of Xiangya School of Medicine, Central South University Changsha, Hunan 410013, China; ^3^NHC Key Laboratory of Carcinogenesis, Hunan Cancer Hospital and The Affiliated Cancer Hospital of Xiangya School of Medicine, Central South University, Changsha, Hunan 410013, China; ^4^Cancer Research Institute, Basic School of Medicine, Central South University, Changsha, Hunan 410011, China; ^5^Department of Neurology, Xiangya Hospital, Central South University, Changsha, Hunan 410008, China; ^6^Department of Oncology, The Second Xiangya Hospital, Central South University, Changsha, Hunan 410008, China

## Abstract

**Background:**

Lung cancer remains the leading cause of cancer death worldwide, and the most subtype is lung adenocarcinoma (LUAD). Tumor-infiltrating immune cells (TIICs) greatly impact the prognosis of LUAD. Tumor necrosis factor–like weak inducer of apoptosis (TWEAK), signal via its receptor fibroblast growth factor-inducible 14 (Fn14), dysregulates immune cell recruitment within tumor environment, thus promoting the progression of autoimmune diseases and cancer. We aimed to explore its role in LUAD.

**Methods:**

The expression level of TWEAK was explored in Tumor Immune Estimation Resource 2.0 (TIMER2.0) and Oncomine databases. The Tumor Immune Dysfunction and Exclusion (TIDE) and Lung Cancer Explorer (LCE) databases were applied to evaluate the survival in correlation to TWEAK expression. TIICs were assessed with TIMER2.0 and TIDE datasets. The expression of TWEAK protein was detected in LUAD cell lines and also in tissue samples from LUAD patients via western blotting or combination with immunochemistry.

**Results:**

Our results showed that TWEAK was downregulated in LUAD tumors compared to normal tissues in TIMER2.0, Oncomine, cell lines, and clinical specimens. Poor survival was uncovered in lower TWEAK expression of LUAD patients in LCE (meta − HR = 0.84 [95% CI, 0.76-0.92]) and TCGA (Continuous Z = −1.97, *p* = 0.0486) and GSE13213@PRECOG (Continuous Z = −4.25, *p* = 2.12e − 5) in TIDE. Multiple tumor-infiltrating immune cells (TIICs) were found closely correlated with TWEAK expression in LUAD, especially hematopoietic stem cell (Rho = 0.505, *p* = 2.78e − 33), common lymphoid progenitor (Rho = −0.504, *p* = 3.79e − 33), and myeloid-derived suppressor cells (MDSCs) (Rho = −0.615, *p* = 1.36e − 52).

**Conclusion:**

Lower level of TWEAK was linked with poor survival and aberrant recruitment and phenotype of TIICs in LUAD, which might motivate immune escape and weaken the effects of immunotherapy.

## 1. Introduction

Lung cancer is a deadly cancer with the highest morbidity and mortality around the world, among which lung adenocarcinoma (LUAD) is the most common pathological type. Currently, the prognosis of LUAD is still not satisfying, and the traditional treatments (including surgery, radiotherapy, and chemotherapy) are limited to a subset of patients with partial remission. The emerging immunotherapy has achieved encouraging results in certain patients, but the prognosis of LUAD treated with immunotherapy was still varied even in the same TNM (Tumor, regional lymph Node, Metastasis) stage.

Tumor necrosis factor–related weak inducer of apoptosis (TWEAK), also termed TNFSF12, is located in chromosomal 17p13.1. TWEAK encodes many cytokines which is widely distributed in normal tissues and produces a variety of functions in cancer through combing with Fn14 (TNFRSF12A) such as angiogenesis, proliferation, apoptosis, fibrosis, and epithelial-mesenchymal transform (EMT) [1–3]. Tumor necrosis factor-related weak inducer of apoptosis (TWEAK), also known as TNFSF12, is located on chromosome 17p13.1 and is a member of the TNF superfamily. TWEAK encodes a variety of cytokines, is widely distributed in normal tissues, and binds in cancer by binding to a type I transmembrane protein whose unique receptor, fibroblast growth factor-inducible 14 (Fn14, TNFRSF12A), has so far been reported producing multiple functions by activating the tumor necrosis factor receptor–associated factor (TRAF) signaling pathway and the nuclear factor kappa B (NF-*κ*B) signaling pathway, such as angiogenesis, proliferation, apoptosis, fibrosis, and epithelial-mesenchymal transition (EMT) [1–3]. TWEAK is also the only ligand that binds to Fn14. Recent studies indicate that the aberrant TWEAK/FN14 pathway was engaged in some autoimmune diseases. TWEAK inhibits T helper 1 cells in the innate immune system by hindering IFN-*γ* and IL-12. Mutant TWEAK causes the lack of antibody by inhibiting the survival of B cells, and TWEAK inhibition can produce an antitumor effect through its regulation on macrophages [4, 5]. Therefore, TWEAK mediates crucial innate and adaptive immune pathways by modulating the function of various TIICs and shows an impact on the efficacy of immunotherapy and compound the prognosis of cancer patients [6–8].

In the present study, we investigated the TWEAK expression in LUAD in TIMER2.0 and TIDE databases and assessed the effect of TWEAK on the survival via TIDE and LCE databases. The relation between TWEAK and TIICs was explored in TIMER2.0 and TIDE. The results shown that low TWEAK expression indicates poor prognosis in LUAD and correlated with various TIICs, possibly due to the defective TWEAK/FN14 pathway.

## 2. Material and Methods

### 2.1. The Expression Profiles of TWEAK

The expression levels of TWEAK in cancers were explored from Tumor Immune Estimation Resource 2.0 (TIMER2.0) and Oncomine databases. TIMER2.0 (https://timer.cistrome.org) which based on a deconvolution method is a comprehensive web server which provides tumor-infiltrating immune cell (TIIC) information from gene expression profiles from The Cancer Genome Atlas (TCGA) [9–11]. Oncomine (https://www.oncomine.org/resource/login.html) is an integrated tool to analyze and validate gene expression and targets [12].

### 2.2. Prognostic Features of TWEAK

The survey of survival information of LUAD patients was carried out though Tumor Immune Dysfunction and Exclusion (TIDE) and Lung Cancer Explorer (LCE) databases. The TIDE database was used to speculate on the functions of genes regulating LUAD immunity and to comprehensively analyze the immune evasion mechanism of immune dysfunction and rejection to LUAD, so as to effectively predict the effect of immune checkpoint inhibition therapy [13]. LCE (https://lce.biohpc.swmed.edu/) is a powerful website to analyze gene expression and related clinical features in lung cancer [14].

### 2.3. The Correlation between TWEAK and TIICs

To analyze the association between TWEAK and TIICs, TIMER2.0 and TIDE databases were analyzed in this study. The relevance to TIICs (such as B cell, CD4+ T cell, CD8+ T cell, neutrophil, macrophage, and dendritic cell) was carried out via the immune-gene module in TIMER2.0 and query gene module in TIDE (Cytotoxic T lymphocytes, CTL), and all the results from TIMER2.0 were adjusted with purity.

### 2.4. PPI Network Analysis

To analyze the protein-protein interaction (PPI) network of TWEAK, the STING database was utilized. The STING database (https://string-db.org/) is a database including PPI networks from more than 24584628 proteins of 5090 organisms [15].

### 2.5. Cell Lines and Cell Culture

The human normal lung epithelial cell line HBE and NSCLC cell lines A549, H1299, H358, SPCA1, PC9, HCC827, and H1993 were purchased from the Cell Biology of Chinese Academy of Science (Shanghai, China). The cells were cultured in RPMI-1640 (Gibco, USA) supplemented with 10% fetal bovine serum (BIOIND, Israel), 100 *μ*g/ml streptomycin, and 100 U/ml penicillin (Gibco, USA) at 37°C in a humidified 5% CO_2_. The cells were passaged every 2-3 days by 0.25% trypsin (Gibco) and not cultured for more than 3 months.

### 2.6. Western Blotting

TWEAK and GAPDH were purchased from ImmunoWay (ImmunoWay Biotechnology Company, Plano, TX). The cells and tissue samples (Department of Thoracic, Xiangya Hospital of Central South University) were harvested and lysed with RIPA protein extraction reagent (Thermo Scientific, USA) supplemented with protease inhibitor cocktail. Approval by the Xiangya Hospital of Central South University Institutional Research Ethics Committee was obtained prior to collecting the archived tissue The protein concentrations were measured using the BCA assay (Pierce, CA, USA). Equal protein amounts were extracts and loaded per well and separated by electrophoresis on 8-10% SDS-PAGE and transferred on to polyvinylidene fluoride (PVDF) membrane (HyClone Laboratories, Logan, UT, USA). The membranes were blocked for 1 h at room temperature in Tris-buffered saline/0.1% Tween 20 (TBST) containing 5% (wt/vol) nonfat milk and then incubated with primary antibodies in TBST containing 5% (wt/vol) nonfat milk at 4°C overnight. The membranes were then incubated with an appropriate secondary antibody coupled to horseradish peroxidase 1 h at 37°C, and the proteins were detected by Luminata Forte western HRP substrate (Millipore, Billerica, MA, USA). Anti-GAPDH levels were detected for normalization.

### 2.7. Immunohistochemistry

Protein expression detected by IHC was performed on LUAD pathological sections. We obtained formalin-fixed, paraffin-embedded recurrent LUAD specimens (40 patients) from the Department of Pathology, Xiangya Hospital of Central South University and prepared tissue sections (5 *μ*m). Patient characteristics are summarized in Supplementary Table S1. The specimens were immunostained using the UltraVision Quanto horseradish peroxidase detection system (Thermo Fisher Scientific). After routine deparaffinization with a series of xylene and alcohols, antigen retrieval was performed using 90% formic acid. Slides were then rinsed with distilled H_2_O and wash buffer. Endogenous peroxidase activity was blocked with H_2_O_2_ solution (TA-125-HP, Thermo Fisher Scientific) for 10 min prior to incubation with a rabbit anti-TWEAK monoclonal antibody (ImmunoWay Biotechnology Company, Plano, TX) at 1 : 100 for 60 min at room temperature. The primary antibody signal was developed with Quanto detection reagents and 3,3′-diaminobenzidine chromogen as per the manufacturer's instructions. Virtual slides were produced by scanning the immunohistochemical (IHC) glass slides using the Aperio CS2 digital pathology scanner (Leica Biosystems). Digital quantitative analysis of TWEAK immunoreactivity in cells was performed by an experienced pathologist in a blinded manner with Aperio ImageScope software v12.2.2.5015 (Leica Biosystems) using a customized positive pixel count algorithm. Stain intensity values are provided as a scoring system for each chromophore comprised of staining intensity and extensiveness captured the outcome: 0, negative; 1, weak; 2 moderate; and 3, strong.

### 2.8. Statistical Analysis

The expression of TWEAK was calculated by the Wilcoxon test, and the purity-adjusted Rho between TWEAK and TIICs was computed by Spearman's correlation coefficient in TIMER2.0. For the TWEAK IHC staining and the western blot signal quantitation results, statistical analysis was performed using 2-way ANOVA and *X*^2^ test. *p* values <0.05 were considered significant.

## 3. Results

### 3.1. The Expression of TWEAK Was Decreased in Tumor Area

**Figure 1:**
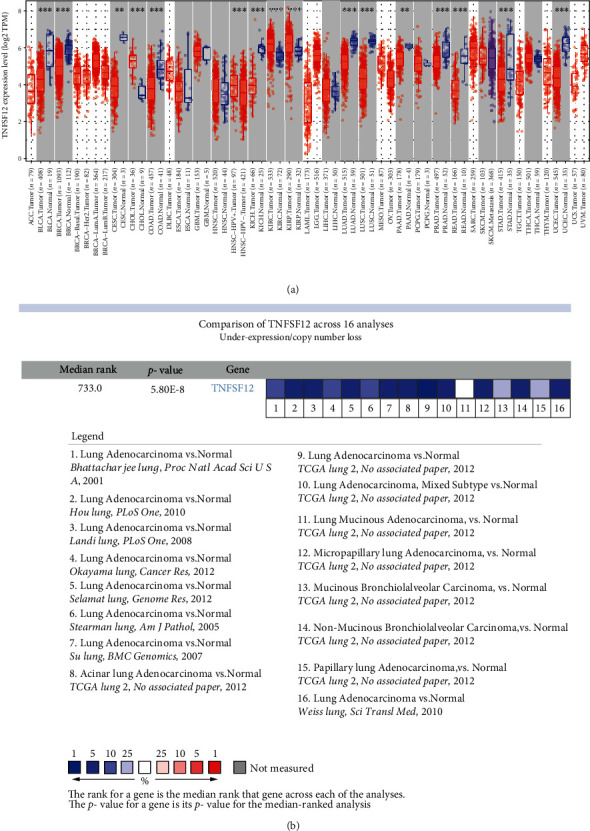
The expression status of TWEAK from TIMER2.0 (a) and Oncomine (b). Note: ^∗^*p* value <0.05; ^∗∗^*p* value <0.01; and ^∗∗∗^*p* value <0.001.

To explore the expression of TWEAK in LUAD, TIMER2.0 and Oncomine databases were used. From TIMER2.0, TWEAK expression was drownregulated in LUAD than normal tissues (Figure 1(a)). Similar results were obtained in 15/16(93.75%) datasets from Oncomine (Figure 1(b)).

### 3.2. Lower Level of TWEAK Correlated to Shorter OS

**Figure 2:**
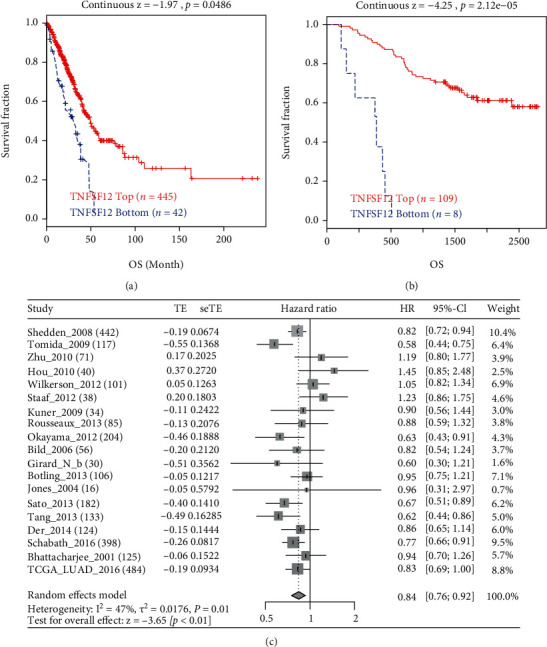
Prognostic value of TWEAK expression in LUAD. TCGA (a), GSE13213@PRECOG, (b) and meta-analyses (c).

In order to explore the overall survival (OS) of LUAD patients according to TWEAK expression, the TIDE and LCE databases were analyzed. From TIDE, poorer OS was greatly relevant to lower TWEAK in TCGA (Continuous Z = −1.97, *p* = 0.0486) (Figure 2(a)) and GSE13213@PRECOG (Continuous Z = −4.25, *p* = 2.12e − 5) (Figure 2(b)). Besides, similar outcomes were obtained from the meta-analysis part of LCE (meta − HR = 0.84 [95% CI, 0.76-0.92]) (Figure 2(c)).

### 3.3. TWEAK Was Highly Related to TIICs

**Figure 3:**
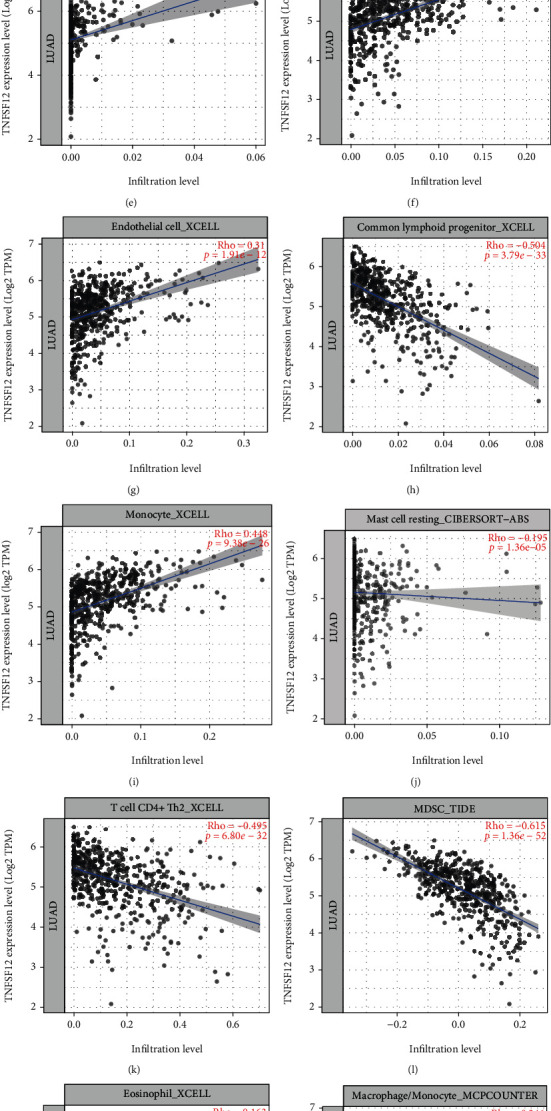
The relationships between TWEAK expression and TIICs in LUAD.

To explore the relationship between TWEAK expressive and TIICs, the TIMER2.0 and TIDE databases were explored (Figure 3). It was revealed from the TIMER2.0 that TWEAK was negatively associated with common lymphoid progenitor, MDSC, mast cell resting, and T cell CD4+Th2, while it was positively related to hematopoietic stem cell, granulocyte-monocyte progenitor, cancer-associated fibroblast, cancer-switched memory B cell, common myeloid progenitor, T cell NK, endothelial cell, monocyte, eosinophil, and macrophage/monocyte. Besides, by analyzing in TIDE, CTLs also were positively correlated with TWEAK in TCGA (*r* = 0.144, *p* = 0.00142) and GSE13213@PRECOG (*r* = 0.255, *p* = 0.00553). Among these TIICs, hematopoietic stem cell, common lymphoid progenitor, and MDSC were the most closed TIICs (|Rho| > 0.5).

### 3.4. The PPI Network of TWEAK

**Figure 4:**
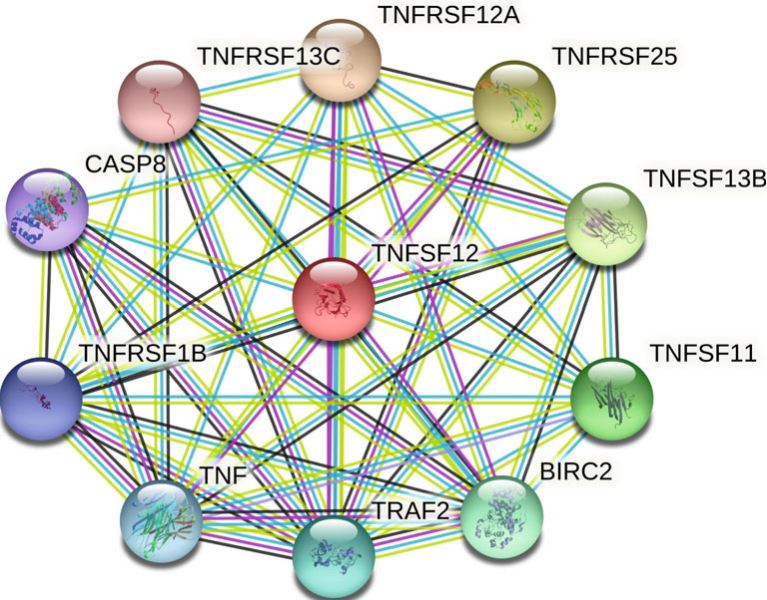
The PPI network of TWEAK. Different color lines: blue: from curated databases; purple: experimentally determined; green: gene neighborhood; black: coexpression; and lavender: protein homology.

To explore the downstream targets of TWEAK, the STRING database was applied (Figure 4). From STRING analyses, we found that many genes had intertwined relationships with TWEAK, including TNFRSF12A, TNFRSF25, TNFSF13B, TNFSF11, BIRC2, TRAF2, TNF, TNFRSF1B, TNFRSF13C, and CASP8.

### 3.5. The Expression of TWEAK in LUAD Cell Lines and Tissues

**Figure 5:**
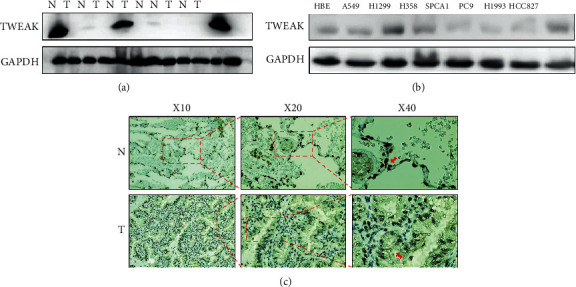
TWEAK expression in (a) normal lung epithelium tissue (N) and LUAD tissue (T), (b) normal lung epithelium cell line and LUAD cell line (detected by WB), (c) normal lung epithelium tissue (N), and LUAD tissue (T) (detected by WB and IHC).

In order to study the expression of TWEAK in LUAD, first, we tested the expression of TWEAK in fresh lung cancer tissues and normal adjacent tissues (See Supplementary Figure S1 for original results). We found that in 6 pairs of samples, TWEAK was strongly positive in 3 pairs of normal lung tissues (Figure 5(a)). Subsequently, we used western blot analysis to detect normal lung epithelial cell lines HBE and NSCLC cell lines A549, H1299, H358, SPCA1, PC9, HCC827, and H1993, among which lung adenocarcinoma cell lines are A549, H1299, SPCA1, and H1993. The results show that the expression of TWEAK in normal lung epithelial cell lines is relatively higher than its expression in lung adenocarcinoma cell lines, but the expression of TWEAK in other nonsmall cell lung cancers (non-LUAD) is higher than that in normal lung epithelial cell lines (Figure 5(b)). At the same time, we found that the TWEAK protein expression of H1299 is higher than that of HBE. Because the characteristic of the H1299 cell line is p53(-), we speculate that the expression of TWEAK may be related to lymphocyte infiltration and lymph node metastasis. However, more research is needed to explain this issue in the future.

Next, we reviewed 40 LUAD pathological specimens and used IHC analysis to detect the expression of TWEAK in the specimens. The clinicopathological characteristics of the LUAD patients are summarized in Supplementary Table 1. There were 26 female and 14 male patients with a median age of 54 years (range, 36-77 years). Histopathologic diagnosis included the following: well differentiated (*n* = 7, 17.5%), moderately differentiated (*n* = 27, 67.5%), and poorly differentiated (*n* = 6, 15%) tumors. Postoperative staging evaluation demonstrated stage I disease in 14 patients, stage II disease in 6 patients, stage III disease in 19 patients, and stage IV disease in 1 patient. We further detect the TWEAK protein expression in LUAD and the correlation with clinicopathological parameters. In the present study, all of the tumor sections were classified as TWEAK-positive as detected by IHC and positive staining was mainly located in the nucleus (Figure 5(c)). In addition, we found that TWEAK stains deeply (+++) in normal lung epithelial cells, while staining is relatively light (+) in LUAD epithelium. Deep staining of nuclei also appeared in the inflammatory cells of LUAD, suggesting that the expression of TWEAK is also related to inflammation [16]. The correlation of TWEAK expression with clinicopathological parameters was then investigated. TWEAK expression was significantly associated with differentiation, pTNM stage, primary tumor size, lymph node metastasis, and tumor location. No significant relationship was noted between TWEAK 19 expression and gender, age, smoking history, and histological type (Table 1).

## 4. Discussion

Lung cancer is still the most severe threat to the population around the world due to its high mortality [17]. LUAD is the majority subtype among lung cancer. Immunotherapy has opened a new field for LUAD treatment because of its comparatively higher tolerance and prolonged effectiveness with possible tumor clearance compared with traditional chemotherapy administration. But the act of immunotherapy somewhat depends on the function of immune cells within tumor itself or around tumor microenvironment.

TWEAK is a type II transmembrane protein, belonging to the member of the tumor necrosis factor superfamily (TNFSF) ligands. It plays an important role in the development of cancer through multiple ways including TRAF and NF*ĸ*B pathways by combining with FN14 [18], such as inflammation [16], proliferation and/or apoptosis of cancer cells, angiogenesis, and epithelial-mesenchymal transform (EMT) [19].

Our study revealed that lower expression of TWEAK was connected with worse prognosis. In TIMER2.0 and Oncomine databases, the results showed the decreased TWEAK expression in LUAD tumors compared to normal tissues. Further investigation based on TIDE and LCE suggested that shorter survival appeared in poorer expression of TWEAK in LUA. Therefore, low TWEAK expression was an inferior prognostic biomarker of LUAD.

Besides, less TWEAK is likely to be related with the infiltrations of TIICs in LUAD. According to the TIDE, the CTL was positively related with TWEAK in TIDE. Furthermore, in TIMER2.0, we found that TWEAK shown up in a subtractive relevance to common lymphoid progenitor, MDSC, mast cell resting, and T cell CD4+ Th2 and in a positive correlation with hematopoietic stem cell, granulocyte-monocyte progenitor, cancer-associated fibroblast, cancer-switched memory B cell, common myeloid progenitor, T cell NK, endothelial cell, monocyte, eosinophil, and macrophage/monocyte, especially hematopoietic stem cell, common lymphoid progenitor, and MDSC.

Existing researches can be used to help decipher our findings of the role of TWEAK. Although TWEAK is upregulated in many tumors, lower TWEAK level was reported in squamous cervical carcinoma [20], endometrial cancer [21], NSCLS [22], and glioblastoma [23]. Furthermore, poor survival with downregulated TWEAK was proved in head and neck cancer and squamous cervical carcinoma [20, 24]. However, it has not been observed that TWEAK had an impact on the survival in NSCLC.

The poor survival of low TWEAK expression patients might connect with various TIICs. Previous studies found that TWEAK tends to be expressed in a variety of immune cell regions (including dendritic cells, circulating NK cells, and resting and activated monocytes [25]). Many studies have confirmed that TWEAK could induce the death of cancer cells through TWEAK/FN14 pathways, such as apoptosis, necrosis, and indirect cell death [4, 26]. TIICs could also inhibit the survival of cancer cells by surveillance and cytotoxicity [27] of the immune system via TWEAK. For example, macrophages could induce apoptosis by motivating CD4+T Cell through TWEAK pathways [28], whereas longtime of immune infiltrating may transform lesions into the status of chronic inflammation [29] and induce the apoptosis of TIICs themselves [30], which may arouse an environment of immune escape and failure to immunotherapy [31]. On the other hand, the increased inhibitory TIICs could also contribute to the progression of LUAD patients. For instance, MDSCs help cancer cells escape from the immune system and resistant to immunotherapy [32]. In short, the death of cancer cells which might be derived from TIICs and TWEAK/FN14 could account for the difference of prognosis in LUAD.

**Figure 6:**
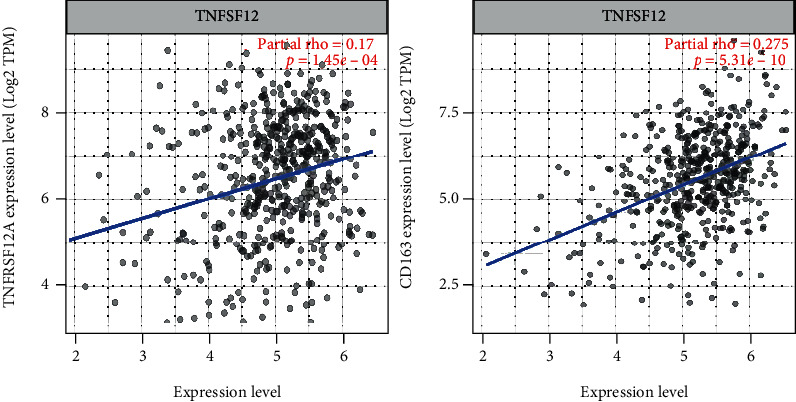
The correlations between TNFSF12 and its receptors. FN14 (a) and CD163 (b).

In addition, it was discovered that TWEAK is positively related with its receptors (FN14, Figure 6(a), and CD163, Figure 6(b)) in TIMER2.0. However, FN14 could accelerate the progression of cancer without the participant of TWEAK [33], which is eccentric to the effects of TWEAK in this study. The reported another receptor, CD163, which appeared on the macrophages and monocytes which could promote cell proliferation [34], was also observed with poor survival in cancer [35]. These erratic phenomena need to be clarified and verified.

Since TWEAK is expressed in many types of solid tumors, it exhibits unprecedented potential clinical application value. A new type of human TWEAK receptor antibody (TweakR, Fn14, TNFRSF12A, and CD266) PDL192 was found to directly inhibit tumor cell growth and antibody-dependent cytotoxicity in a variety of mouse xenograft models, showing strong antitumor effects active [36]. Another TWEAK receptor antibody, RG7212, moved from the laboratory to clinical trials. RG7212 inhibits tumor growth by inhibiting tumor cell proliferation and survival signals, enhancing the host's antitumor immune response, but depends on the positive expression of Fn14 [37–39].

We found from the Kaplan-Meier database that the high expression of TweakR (Fn14) suggested poor OS (*n* = 719, *p* = 0.0009), but the expression of TWEAK was not significantly correlated with OS in LUAD patients (*n* = 719, *p* = 0.2721). Lab evidence suggests that low serum levels of TWEAK may be one of the characteristics of NSCLC [40], and TWEAK/Fn14 induce NSCLC survival rate and treatment response by Mcl-1mediated [41]. Interestingly, among LUAD patients receiving chemotherapy, those with high TWEAK expression levels had poorer OS (*n* = 36, *p* = 0.02), suggesting that TWEAK is related to chemotherapy resistance. In the study of ovarian cancer, Fn14 seems to be able to overcome the resistance of chemotherapy drugs [42] and in gliomas, it is highly expressed in PDX of resistant patients [43]. This seemingly contradictory phenomenon undoubtedly indicates that TWEAK has potentially more unique and unexpected functions.

TWEAK is a type II transmembrane protein, but it can be cleaved by furin to produce soluble cytokines. Therefore, both membrane-anchored and soluble TWEAK can bind to Fn14 [44–46]. TWEAK is a glycoprotein with three parts, including a C-terminal extracellular domain, a transmembrane domain, and an N-terminal intracellular domain. Fn14 contains an extracellular domain that binds to TWEAK and a cytoplasmic tail necessary for signal transduction [47, 48]. Activation of TWEAK/Fn14 signaling triggers intracellular signaling cascades that include regulation of cell death (apoptosis or necrosis), proliferation, differentiation and migration, triggering of angiogenesis, and induction of inflammatory cytokine expression.

TWEAK/Fn14 signaling pathway is involved in tumor pathogenesis. It plays an important role in the growth, invasion, and migration of tumor cells. Furthermore, TWEAK/Fn14 activation triggers downstream signaling to regulate several key events related to tumor inflammation, angiogenesis, and EMT. Given their high tumor-related expression and multiple roles, TWEAK and Fn14 are considered two attractive targets for tumor therapy. Therefore, many drugs targeting TWEAK or Fn14 have been developed by researchers around the world in recent years (Table 2), and some TWEAK and Fn14 targeting drugs have been tested in preclinical trials and showed effective results. They exert antitumor effects through three pathways: neutralize soluble TWEAK, block Fn14 signaling, and directly kill Fn14-positive tumor cells. In the future, we should focus on basic and translational research on the TWEAK-Fn14 axis, which will be a suitable molecular target for the development of new tumor therapies; at the same time, more preclinical studies are needed to explore the safety of TWEAK/Fn14 in clinical practice effective treatment.

## 5. Conclusions

In this study, we found that lower TWEAK was related to poor prognosis and TIICs in LUAD. And decreased TWEAK was correlated with multiple immune cells in the tumor region. Intimate relationship between FN14 and TWEAK indicated that TWEAK/FN14 pathway possibly plays an important role in the survival of LUAD, but the underlying mechanism needs to be further explored.

## Figures and Tables

**Table 1:** Association of TWEAK expression with clinicopathological features in LUAD specimens.

Variables	Number	Tweak expression			
		+	++	+++	*p* value
Sex		23	12	5	
Male	14	7	5	2	0.73
Female	26	16	7	3	
Age (years)					
<58	19	8	7	4	0.28
≥58	21	15	5	1	
Smoking history					
Smoker	19	8	10	1	0.62
Nonsmoker	21	15	2	4	
Differentiation					
Well	7	1	2	4	<0.05 ^∗^
Moderate	27	17	9	1	
Poor	6	5	1	0	
pTNM stages					
I-II	19	16	2	1	<0.05 ^∗^
III-IV	21	7	10	4	
Primary tumor size(cm)					
<4 cm	15	8	4	3	0.19
≥4 cm	25	15	8	2	
Lymph node metastasis					
Yes	26	16	10	0	<0.05 ^∗^
No	14	7	2	5	
Tumor location					
Central	6	3	2	1	<0.05 ^∗^
Peripheral	34	20	10	4	

^∗^ *p* value of *X*^2^ test is shown. pTNM: pathological tumor/node metastasis.

**Table 2:** TWEAK/Fn14 targeting therapeutic agents against cancers.

Target	Agent	Type of agent
Tweak [49–52]	RG7212 (RO5458640)	Neutralizing mAb
	Fn14-TRAIL (kahr-101)	Signal converter protein
Fn14 [53–61]	BIIB036 (P4A8)	Agonistic mAb
	I8DI	Agonistic mAb
	PDLI92	Agonistic mAb
	ITEM4-rGel	Immunotoxin conjugate
	hSGZ	Immunotoxin fusion protein
	Granzyme (GrB)-TWEAK and GrB-Fc-IT4	GrB-containing fusion protein
	Anti-Fn14 antibody conjugated nanoparticles	Drug-loaded nanoparticles

TRAIL: Tumor necrosis factor-related apoptosis-inducing ligand; mAb: Monoclonal antibody.

## Data Availability

The data used to support the findings of this study are available from the corresponding authors upon request.

## Supplementary Materials

Supplementary Materials Figure S1: the original data of TWEAK and GAPDH expression in (A, B) normal lung epithelium tissue (C, D) and normal lung epithelium cell line and LUAD cell line (detected by WB). Table S1: characteristics of LUAD patients.
