# Supplementary material for: Lower Expression of TWEAK is Associated with Poor Survival and Dysregulate TIICs in Lung Adenocarcinoma
Source: Dis Markers. 2022 Jun 6;2022:8661423. doi: 10.1155/2022/8661423 (PMC9192298; doi:10.1155/2022/8661423)
Supplement: Supplementary Materials — Figure S1: the original data of TWEAK and GAPDH expression in (A, B) normal lung epithelium tissue (C, D) and normal lung epithelium cell line and LUAD cell line (detected by WB). Table S1: characteristics of LUAD patients. [file 8661423.f1.zip › Table S1.docx]

Table S1. Characteristics of LUAD patients.

|  | LUAD (n=40) |
| --- | --- |
| Age (years) | 63.7±9.33 |
| Sex |  |
| Male | 14 |
| Female | 26 |
| TNM stage |  |
| 0-2 | 19 |
| 3A+3B | 11 |
| 4 | 10 |
| Metastasis |  |
| Yes | 26 |
| No | 14 |
